# Semisynthesis and insecticidal activity of some novel fraxinellone-based thioethers containing 1,3,4-oxadiazole moiety

**DOI:** 10.1098/rsos.171053

**Published:** 2017-12-13

**Authors:** Yong Guo, Xiaoguang Wang, Jiangping Fan, Qian Zhang, Yi Wang, Yi Zhao, Mengxing Huang, Ming Ding, Yanbing Zhang

**Affiliations:** 1 Key Laboratory of Advanced Drug Preparation Technologies, Ministry of Education, School of Pharmaceutical Sciences, Zhengzhou University, Zhengzhou, 450001, Henan Province, People's Republic of China; 2 College of Agriculture, Shanxi Agriculture University, Taigu 030801, Shanxi Province, People's Republic of China

**Keywords:** fraxinellone, 1,3,4-oxadiazole, structural modification, insecticidal activity, structure–activity relationships

## Abstract

Two series of novel fraxinellone-based thioethers containing 1,3,4-oxadiazole moiety were prepared as insecticidal agents against the oriental armyworm, *Mythimna separata* Walker. The structural assignment was based on the spectroscopic and X-ray analysis data. Among all the target compounds, compounds **4b**, **4k**, **5b**, **5j** and **5k** exhibited more potent insecticidal activity with final mortality rates (FMRs) of more than 65%, especially **4k** with the FMR of 75.9%, when compared with toosendanin. Some interesting results of structure–activity relationships are also discussed.

## 1. Introduction

The oriental armyworm (*Mythimna separata* Walker, Lepidoptera: Noctuidae), a typical long-distance migratory insect, is one of the most serious pests of cereal crops in countries including China, India, Australia and New Zealand [1,2]. Seasonal outbreaks of this pest can cause significant economic damage to cereal crops in China and other countries [3]. A recent outbreak of *M. separata* has been reported in northeast and central China during 2012, which caused losses of approximately 10 million acres of crops [4]. Synthetic chemical pesticides play a crucial role in agriculture with the characteristics of high-efficiency, quick-fix and broad-spectrum insecticides. Although they have been extensively used to control insect pest outbreaks, the overuse and improper application of synthetic chemical pesticides over the years has resulted in enhancement of pest resistance, environmental problems and negative impacts on human health [5–7]. Hence the discovery and development of effective, selective and eco-friendly pesticides is necessary in the future.

**Figure 1.**
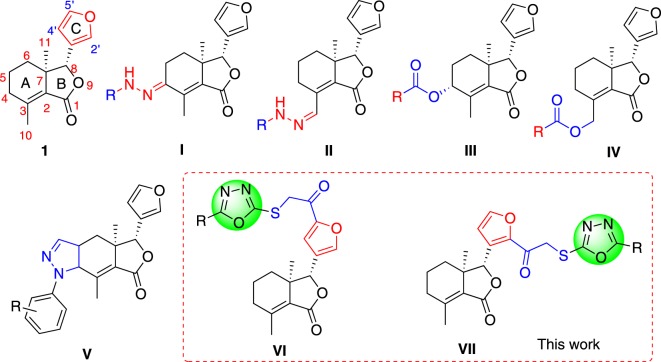
Chemical structures of fraxinellone (**1**) and its derivatives (**I**–**VII**).

Fraxinellone (**1**, figure 1), a naturally occurring degraded limonoid, isolated from *Fagaropsis glabra*, [8] *Dictamnus albus*, [9] *Melia azadarach* [10] and *Dictamnus dasycarpus*, [11] exhibits a variety of interesting activities both in the fields of medicinal chemistry and agrochemistry, such as anti-inflammatory [12], vascular relaxing activity [13] and insecticidal activity [14–16]. The total synthesis of fraxinellone can be easily achieved, which has been reported early in 1972 [17]. Previously, we have studied the insecticidal activity of some fraxinellone-based hydrazones and esters [18,19] (**I**–**IV**, figure 1) modified at the C-4 or C-10 position in the A ring of fraxinellone, and *N*-phenylpyrazole fraxinellone hybrid compounds [20] (**V**, figure 1), and found some compounds against *M. separata* displayed higher insecticidal activity than positive control toosendanin. To the best of our knowledge, little attention has been paid to the introduction of active *N*-heterocyclic moieties on the furyl-ring of fraxinellone as insecticidal agents. 1,3,4-Oxadiazoles are an important class of *N*-heterocyclic compounds with a wide range of biological activities [21] including antimicrobial, analgesic, anticancer activities, especially insecticidal and herbicidal activities [22,23]. In a continuation of our programme aimed at the development of fraxinellone-based insecticidal agents, herein we prepared two series of novel fraxiellone-based thioethers containing 1,3,4-oxadiazole moiety (**VI** and **VII**, figure 1) as insecticidal agents against *M. separata*.

## 2. Experimental

### 2.1. Instrument and materials

**Scheme 1.**
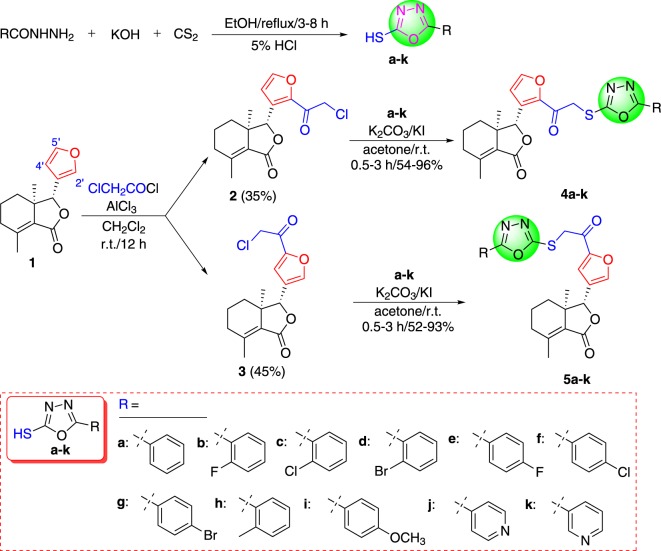
Synthetic route for the preparation of fraxiellone-based thioethers containing 1,3,4-oxadiazole moiety (**4a**–**k** and **5a**–**k)**.

The intermediate 2-mercapto-5-aryl-1,3,4-oxadiazoles **a**–**k** (scheme 1) were synthesized as previously reported [24]. Other reagents were of analytically grade and purchased from commercial resources. Fraxinellone (**1**) was isolated from *Dictamnus dasycarpus* and its purity was more than 99% as measured with reverse phase high-performance liquid chromatography (RP-HPLC). Analytical thin-layer chromatography (TLC) and preparative thin-layer chromatography (PTLC) were prepared by silica gel plates using silica gel GF_254_ (Qingdao Haiyang Chemical Co., Ltd, Qingdao, China). Melting points were determined on a digital melting-point apparatus and were uncorrected (Beijing Tech Instrument Co., Ltd). Optical rotation was measured using an Autopol III automatic polarimeter (Rudolph Research Analytical, NJ, USA). Infrared spectra (IR) were recorded on a PE-1710 FT-IR spectrometer (Perkin-Elmer, Waltham, MA, USA). NMR spectra were obtained in CDCl_3_ on a Bruker Avance (400 MHz) spectrometer using tetramethylsilane (TMS) as the internal standard (Bruker, Bremerhaven, Germany). High-resolution mass spectra (HR-MS) were carried out with LTQ FT Ultra instrument (Thermo Fisher Scientific Inc., Waltham, MA, USA).

#### 2.1.1. Data for **1**

White solid, m.p. 113–115°C; IR cm^–1^: 3148, 2930, 1741, 1671, 1607, 1202, 1022; ^1^H NMR (400 MHz, CDCl_3_) *δ*: 7.46 (s, 1H, H-2′), 7.43 (t, *J* = 1.6 Hz, 1H, H-5′), 6.34 (d, *J *= 1.2 Hz, 1H, H-4′), 4.88 (s, 1H, H-8), 2.13–2.31 (m, 2H, H-4), 2,11(s, 3H, H-10), 1.71–1.87 (m, 3H, H-5, 6), 1.42–1.48 (m, 1H, H-6), 0.86 (s, 3H, H-9); MS (ESI), *m/z* (%) 233.05 ([M + H]^+^, 58).

### 2.2. General procedure for synthesis of compounds **2** and **3**

To a stirred suspension solution of AlCl_3_ (1.0 mmol) in dry CH_2_Cl_2_ (5 ml) at RT, chloroacetyl chloride (1.1 mmol) was added. The mixture was then stirred for 10 min, and a solution of compound **1** (1.0 mmol) in dry CH_2_Cl_2_ (5 ml) was added dropwise to the above mixture. When the reaction was complete according to TLC analysis, the reaction mixture was poured into ice water (15 ml) and extracted with CH_2_Cl_2_ (40 ml × 3). The combined organic phase was washed with saturated brine (40 ml), dried over anhydrous Na_2_SO_4_, concentrated *in vacuo*, and then purified by PTLC to give the pure products **2** (35% yield) and **3** (45% yield).

#### 2.2.1. Data for **2**

White solid, yield: 35%, m.p. 96–98°C; [*α*]^20^_D_ = 45 (*c* 4.0 mg ml^–1^, acetone); IR cm^–1^: 2946, 2918, 2872, 1757, 1687, 1472, 1203, 975; ^1^H NMR (400 MHz, CDCl_3_) *δ*: 7.55 (d, *J* = 1.6 Hz, 1H, H-5′), 6.79 (d, *J* = 2.0 Hz, 1H, H-4′), 5.66 (s, 1H, H-8), 4.60–4.72 (m, 2H, –C*H*_2_Cl), 2.19–2.26 (m, 2H, H-4), 2.13 (s, 3H, H-10), 1.77–1.83 (m, 2H, H-5, 6), 1.58–1.63 (m, 2H, H-5, 6), 0.84 (s, 3H, H-11); HRMS (ESI): Calcd for C_16_H_18_O_4_Cl ([M+ H]^+^), 309.0888; found, 309.0888.

#### 2.2.2. Data for **3**

White solid, yield: 45%, m.p. 106–108°C; [*α*]^20^_D_ = –20 (*c* 3.5 mg ml^–1^, acetone); IR cm^–1^: 2956, 2923, 2870, 1737, 1671, 1496, 1235, 908; ^1^H NMR (400 MHz, CDCl_3_) *δ*: 7.68 (s, 1H, H-2′), 7.27 (s, 1H, H-4′), 4.89 (s, 1H, H-8), 4.59 (s, 2H, –C*H*_2_Cl), 2.23–2.34 (m, 2H, H-4), 2.14 (s, 3H, H-10), 1.72–1.86 (m, 3H, H-5, 6), 1.47–1.53 (m, 1H, H-6), 0.84 (s, 3H, H-11); HRMS (ESI): Calcd for C_16_H_18_O_4_Cl ([M + H]^+^), 309.0888; found, 309.0888.

### 2.3. General procedure for synthesis of compounds **4a–k** and **5a–k**

A mixture of the corresponding 2-mercapto-5-aryl-1,3,4-oxadiazole (0.3 mmol), **2** or **3** (0.2 mmol, 61.6 mg), K_2_CO_3_ (0.3 mmol, 41.5 mg) and KI (0.05 mmol, 8.3 mg) in acetone (10 ml) was stirred at room temperature. After the reaction was complete according to TLC analysis, the solvent was removed and the residue was dissolved in CH_2_Cl_2_ and filtered. The filtrate was concentrated *in vacuo* and purified by PTLC to give pure products **4a–k** and **5a–k**. The example data of **4a–d** and **5a–d** are described as follows, whereas the data of other compounds **4e–k** and **5e–k** are shown in the electronic supplementary material.

#### 2.3.1. Data for **4a**

White solid, yield: 54%, m.p. 76–79°C; [*α*]^20^_D_ = 18 (*c* 3.4 mg ml^–1^, acetone); IR cm^–1^: 2935, 1750, 1673, 1587, 1473, 1414, 1355, 1204, 1129, 1046; ^1^H NMR (400 MHz, CDCl_3_) *δ*: 7.97 (dd, *J* = 7.6, 1.2 Hz, 2H, –Ph), 7.60 (d, *J* = 1.6 Hz, 1H, H-5′), 7.47–7.53 (m, 3H, –Ph), 6.83 (d, *J* = 1.2 Hz, 1H, H-4′), 5.67 (s, 1H, H-8), 4.67–4.87 (m, 2H, –COC*H*_2_S–), 2.13–2.26 (m, 5H, H-4, 10), 1.71–1.77 (m, 2H, H-5, 6), 1.55–1.61 (m, 2H, H-5, 6), 0.86 (s, 3H, H-11).

#### 2.3.2. Data for **4b**

White solid, yield: 68%, m.p. 66–68°C; [*α*]^20^_D_ = 11 (*c* 3.6 mg ml^–1^, acetone); IR cm^–1^: 2923, 1752, 1674, 1588, 1495, 1472, 1414, 1204, 1129, 1048; ^1^H NMR (400 MHz, CDCl_3_) *δ*: 7.97–8.01 (m, 1H, –Ph), 7.60 (d, *J* = 1.6 Hz, 1H, H-5′), 7.50–7.55 (m, 1H, –Ph), 7.28 (d, *J* = 8.0 Hz, 1H, –Ph), 7.21 (d, *J* = 8.4 Hz, 1H, –Ph), 6.83 (d, *J* = 1.6 Hz, 1H, H-4′), 5.67 (s, 1H, H-8), 4.67–4.88 (m, 2H, –COC*H*_2_S–), 2.13–2.26 (m, 5H, H-4, 10), 1.73–1.77 (m, 2H, H-5, 6), 1.55–1.63 (m, 2H, H-5, 6), 0.86 (s, 3H, H-11).

#### 2.3.3. Data for **4c**

White solid, yield: 72%, m.p. 81–83°C; [*α*]^20^_D_ = 10 (*c* 4.2 mg ml^–1^, acetone); IR cm^–1^: 2927, 1753, 1673, 1587, 1478, 1414, 1355, 1275, 1204, 1129, 1046; ^1^H NMR (400 MHz, CDCl_3_) *δ*: 7.91 (dd, *J* = 7.6, 1.6 Hz, 1H, –Ph), 7.60 (d, *J* = 1.6 Hz, 1H, H-5′), 7.52–7.54 (m, 1H, –Ph), 7.44 (td, *J* = 7.6, 2.0 Hz, 1H, –Ph), 7.37 (td, *J* = 7.6, 1.2 Hz, 1H, –Ph), 6.83 (d, *J* = 1.6 Hz, 1H, H-4′), 5.67 (s, 1H, H-8), 4.67–4.88 (m, 2H, –COC*H*_2_S–), 2.15–2.26 (m, 2H, H-4), 2.13 (s, 3H, H-10), 1.71–1.77 (m, 2H, H-5, 6), 1.54–1.58 (m, 2H, H-5, 6), 0.86 (s, 3H, H-11).

#### 2.3.4. Data for **4d**

Pale yellow solid, yield: 73%, m.p. 64–66°C; [*α*]^20^_D_ = 5 (*c* 3.7 mg ml^–1^, acetone); IR cm^–1^: 2931, 1752, 1673, 1587, 1475, 1413, 1355, 1259, 1203, 1129, 1046; ^1^H NMR (400 MHz, CDCl_3_) *δ*: 7.84 (d, *J* = 8.4 Hz, 2H, –Ph), 7.63 (d, *J* = 8.4 Hz, 2H, –Ph), 7.60 (d, *J* = 1.6 Hz, 1H, H-5′), 6.83 (d, *J* = 1.6 Hz, 1H, H-4′), 5.66 (s, 1H, H-8), 4.67–4.88 (m, 2H, –COC*H*_2_S–), 2.08–2.26 (m, 2H, H-4, 10), 1.71–1.77 (m, 2H, H-5, 6), 1.54–1.58 (m, 2H, H-5, 6), 0.86 (s, 3H, H-11).

#### 2.3.5. Data for **5a**

White solid, yield: 65%, m.p. 81–83°C; [*α*]^20^_D_ = –2 (*c* 3.2 mg ml^–1^, acetone); IR cm^–1^: 2940, 2920, 1744, 1676, 1474, 1205, 1133, 1048, 1003; ^1^H NMR (400 MHz, CDCl_3_) *δ*: 7.98 (d, *J* = 6.4 Hz, 2H, –Ph), 7.70 (s, 1H, H-2′), 7.47–7.53 (m, 3H, –Ph), 7.29 (s, 1H, H-4′), 4.89 (s, 1H, H-8), 4.73 (s, 2H, –COC*H*_2_S–), 2.17–2.34 (m, 2H, H-4), 2.14 (s, 3H, H-10), 1.83–1.91 (m, 2H, H-5, 6), 1.71–1.78 (m, 1H, H-5), 1.46–1.53 (m, 1H, H-6), 0.85 (s, 3H, H-11).

#### 2.3.6. Data for **5b**

White solid, yield: 87%, m.p. 111–112°C; [*α*]^20^_D_ = –19 (*c* 3.2 mg ml^–1^, acetone); IR cm^–1^: 2935, 1750, 1682, 1618, 1495, 1472, 1388, 1206, 1161, 1047; ^1^H NMR (400 MHz, CDCl_3_) *δ*: 7.97–8.01 (m, 1H, –Ph), 7.71 (s, 1H, H-2′), 7.48–7.55 (m, 1H, –Ph), 7.21–7.30 (m, 3H, H-4′ and –Ph), 4.90 (s, 1H, H-8), 4.73 (s, 2H, –COC*H*_2_S–), 2.17–2.34 (m, 2H, H-4), 2.14 (s, 3H, H-10), 1.84–1.87 (m, 2H, H-5, 6), 1.73–1.77 (m, 1H, H-5), 1.47–1.54 (m, 1H, H-6), 0.85 (s, 3H, H-11).

#### 2.3.7. Data for **5c**

White solid, yield: 79%, m.p. 106–108°C; [*α*]^20^_D_ = –12 (*c* 2.1 mg ml^–1^, acetone); IR cm^–1^: 2930, 1746, 1675, 1599, 1506, 1474, 1466, 1265, 1208, 1169, 1037; ^1^H NMR (400 MHz, CDCl_3_) *δ*: 7.91 (dd, *J* = 7.6, 1.6 Hz, 1H, –Ph), 7.70 (s, 1H, H-2′), 7.53 (d, *J* = 8.0 Hz, 1H, –Ph), 7.44 (td, *J* = 8.0, 2.0 Hz, 1H, –Ph), 7.38–7.41 (m, 1H, –Ph), 7.29 (s, 1H, H-4′), 4.90 (s, 1H, H-8), 4.73 (s, 2H, –COC*H*_2_S–), 2.17–2.34 (m, 2H, H-4), 2.14 (s, 3H, H-10), 1.84–1.87 (m, 2H, H-5, 6), 1.73–1.77 (m, 1H, H-5), 1.46–1.54 (m, 1H, H-6), 0.86 (s, 3H, H-11).

#### 2.3.8. Data for **5d**

Pale yellow solid, yield: 89%, m.p. 131–132°C; [*α*]^20^_D_ = –16 (*c* 3.2 mg ml^–1^, acetone); IR cm^–1^: 2961, 2930, 2862, 1746, 1675, 1600, 1507, 1466, 1393, 1317, 1207, 1169, 1020; ^1^H NMR (400 MHz, CDCl_3_) *δ*: 7.86 (dd, *J* = 7.6, 1.6 Hz, 1H, –Ph), 7.72 (dd, *J* = 8.0, 1.2 Hz, 1H, –Ph), 7.70 (s, 1H, H-2′), 7.42 (td, *J* = 7.6, 1.2 Hz, 1H, –Ph), 7.35 (td, *J* = 7.6, 1.6 Hz, 1H, –Ph), 7.30 (s, 1H, H-4′), 4.90 (s, 1H, H-8), 4.74 (s, 2H, –COC*H*_2_S–), 2.17–2.34 (m, 2H, H-4), 2.14 (s, 3H, H-10), 1.84–1.87 (m, 2H, H-5, 6), 1.73–1.77 (m, 1H, H-5), 1.46–1.54 (m, 1H, H-6), 0.86 (s, 3H, H-11).

### 2.4. X-ray crystallography

The structures of compounds **4e** and **5d** were unambiguously confirmed by X-ray crystallography. Crystallographic data (excluding structure factors) of compounds **4e** and **5d** were deposited at the Cambridge Crystallographic Data Centre (CCDC) with deposition numbers of CCDC 1552786 and 1552787, respectively.

### 2.5. Biological assay

Growth inhibitory activity of compounds **1**–**3**, **4a**–**k** and **5a**–**k** against *M. separata* was evaluated by leaf-dipping method as described previously [19,25]. For each compound, 30 pre-third-instar larvae of same size and level of health (10 larvae per group) were chosen as the tested pests. Solutions of compounds **1**–**3**, **4a**–**k** and **5a**–**k** and toosendanin (used as a positive control) were prepared in acetone at the concentration of 1 mg ml^−1^. The larvae of tested groups were fed with compound-coated leaves (fresh corn leaf discs (1 × 1 cm) were dipped into the corresponding solution for 3 s, then taken out and dried at RT), whereas the blank control group (CK) was fed with acetone alone. Several treated leaf discs were kept in each dish. Once the treated leaves were consumed, the corresponding ones were added to the dish. The experiment was carried out at 25 ± 2°C; relative humidity (RH) 65–80%, and on 12 h/12 h (light/dark) photoperiod. After 48 h, untreated fresh leaves were added to all dishes until the adult emergence. The corrected mortality rate values of the tested compounds were calculated by the following formula: corrected mortality rate (%) = (*T* – *C*) × 100/(1 – *C*); Where *T* is the mortality rate in the treated group, and *C* is the mortality rate of CK.

## 3. Result and discussion

### 3.1. Synthesis

**Figure 2.**
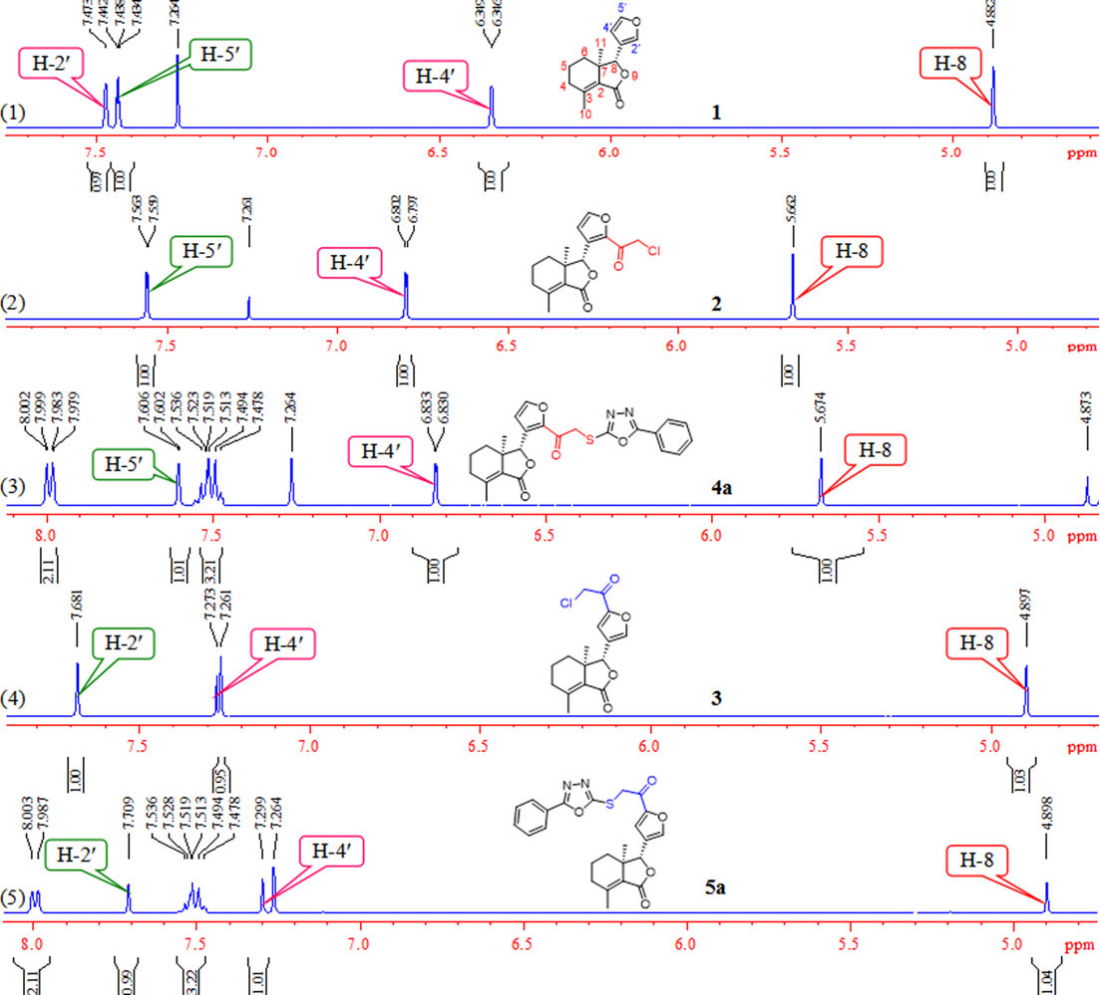
Comparison of partial ^1^H NMR spectra of compounds **1**–**3**, **4a** and **5a**.

As shown in scheme 1, compounds **a–k** were synthesized based on previously reported literature [24]. Using different hydrazides as starting materials, compounds **a–k** were prepared by cyclization reaction of different hydrazides with carbon disulfide in the presence of KOH in EtOH at reflux temperature, followed by acidification with 5% HCl. When fraxinellone reacted with chloroacetyl chloride in the presence of AlCl_3_, the corresponding 2'-chloroacetylfraxinellone (**2**) and 5'-chloroacetylfraxinellone (**3**) were obtained. Subsequently, different 2-mercapto-5-aryl-1,3,4-oxadiazoles (**a**–**k**) reacted with compound **2** or **3** in the presence of K_2_CO_3_/KI in anhydrous acetone at RT to smoothly acquire desired compounds **4a**–**k** and **5a**–**k,** respectively. The structures of all target compounds **4a**–**k** and **5a**–**k** were fully characterized by melting points, IR, optical rotation and ^1^H NMR. Additionally, comparison of partial ^1^H NMR spectra of compounds **1**–**3**, **4a** and **5a** was illustrated in figure 2. It is obvious that the chemical shifts of H-4′ and H-8 of 2'-substituted fraxinellone are very different from 5'-substituted fraxinellone. When chloroacetyl substituted on 2'-position of fraxinellone, the proton of H-4′ was shifted from 6.34 [d, *J* = 1.2 Hz, **1**, figure 2 (1)] to 6.79 [d, *J* = 2.0 Hz, **2**, figure 2 (2)] ppm, and the proton of H-8 was shifted from 4.88 [s, **1**, figure 2 (1)] to 5.66 [s, **2**, figure 2 (2)] ppm; By contrast, when chloroacetyl substituted on 5'-position of fraxinellone, the proton of H-4′ was largely shifted from 6.34 [d, *J* = 1.2 Hz, **1**, figure 2 (1)] to 7.27 [s, **3**, figure 2 (4)] ppm, while the proton of H-8 was slightly shifted from 4.88 [s, **1**, figure 2 (1)] to 4.89 ppm [s, **3**, figure 2 (4)]. The chemical shifts of H-4′ and H-8 of compounds **4a** and **5a** were similar to compounds **2** and **3**, respectively.

**Figure 3.**
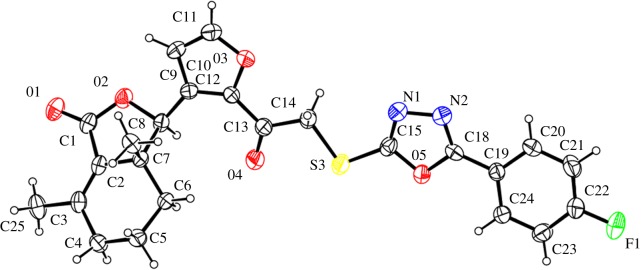
X-ray crystal structure of compound **4e**.

**Figure 4.**
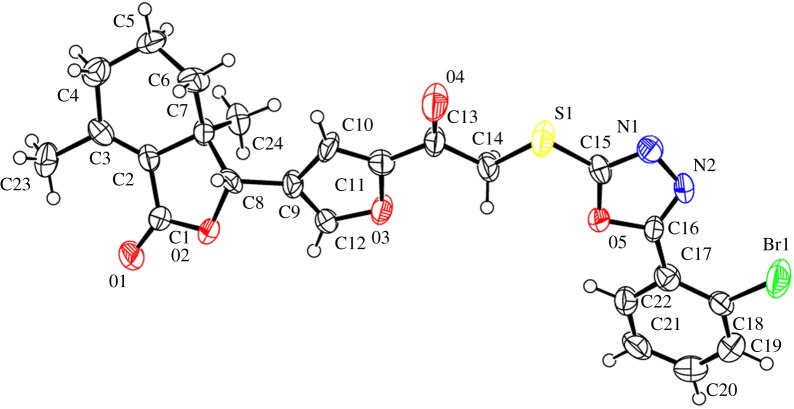
X-ray crystal structure of compound **5d**.

The structures of compounds **4e** and **5d** were further confirmed by X-ray crystallography (figures 3 and 4). As shown in figure 3, to the compound **4e**, 2-mercapto-5-(4-fluorophenyl)-1,3,4-oxadiazole (**e**) linked with chloroacetyl was at the 2'-position on the furyl ring. On the contrary, to the compound **5d** (figure 4), 2-mercapto-5-(2-bromophenyl)-1,3,4-oxadiazole (**d**) linked with chloroacetyl was at the 5'-position on the furyl ring of fraxinellone.

### 3.2. Insecticidal activity

**Figure 5.**
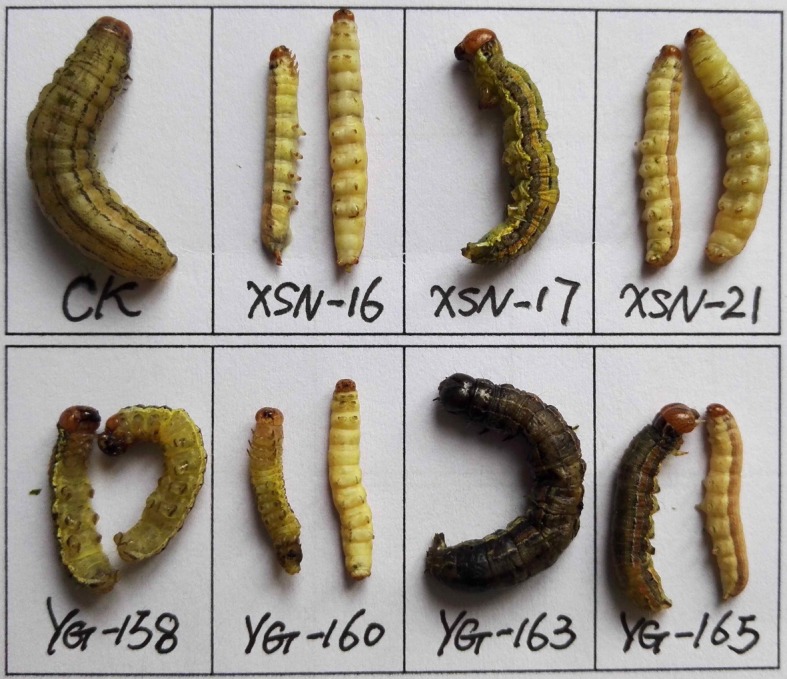
The representative abnormal larvae pictures of **5e** (XSN-16), **4e** (XSN-17), **4b** (XSN-21), **5c** (YG-158), **5d** (YG-160), **5j** (YG-163) and **5k** (YG-165) during the larval period (CK: blank control group).

**Figure 6.**
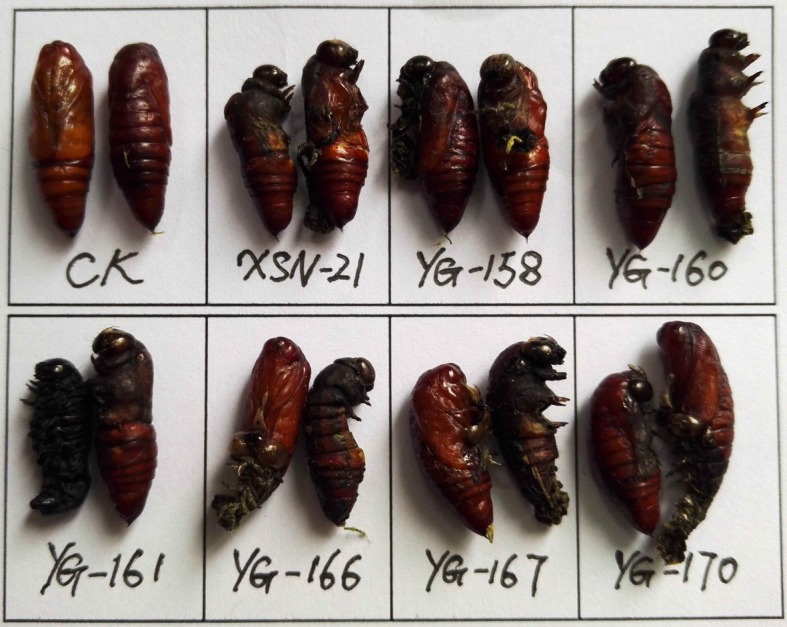
The representative malformed pupae pictures of **4b** (XSN-21), **5c** (YG-158), **5d** (YG-160), **5i** (YG-161), **4a** (YG-166), **4f** (YG-167) and **4d** (YG-170) during the pupation period (CK: blank control group).

**Figure 7.**
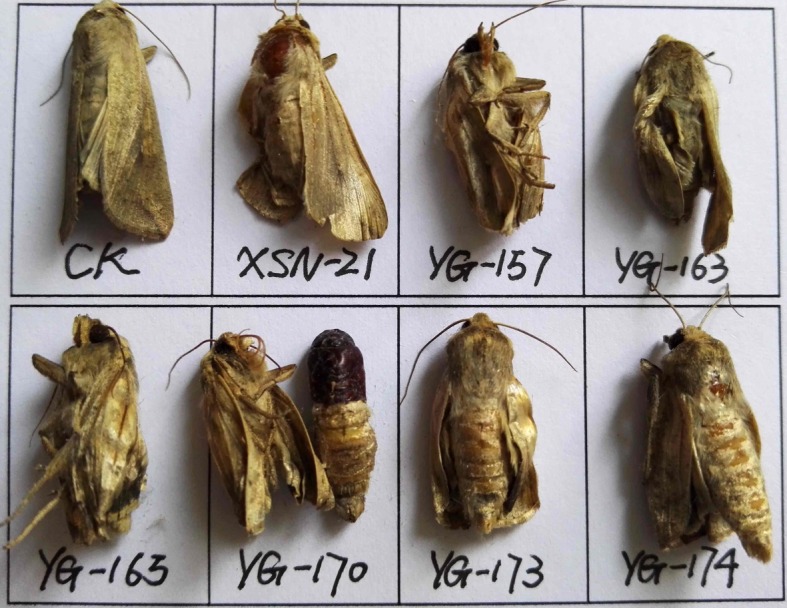
The representative malformed moth pictures of **4b** (XSN-21), **5b** (YG-157), **5j** (YG-163), **5k** (YG-165), **4d** (YG-170), **4j** (YG-173) and **4k** (YG-174) during the emergence period (CK: blank control group).

**Figure 8.**
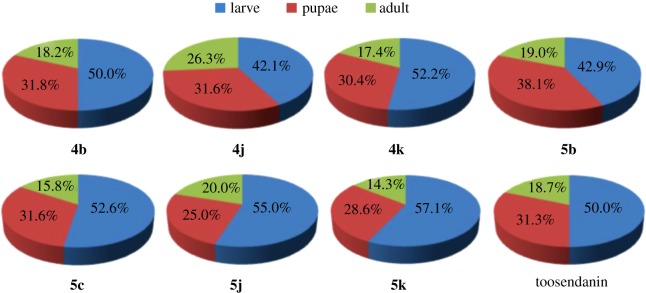
The percentages of FMRs during three different growth periods of compounds **4b**, **4j**, **4 k**, **5b**, **5c**, **5j**, **5k** and toosendanin.

The growth inhibitory activity of compounds **1**–**3**, **4a**–**k** and **5a**–**k** against *M. separata* was tested at 1 mg ml^−1^. Toosendanin, a commercial insecticide derived from *Melia azedarach*, was used as the positive control at 1 mg ml^−1^, and corn leaves treated with acetone alone were used as a blank control. As shown in table 1, compounds **4b**, **4c**, **4j**, **4k**, **5b**–**d**, **5j**, **5k** exhibited higher insecticidal activity than toosendanin and their precursor fraxinellone. For example, the final mortality rates (FMRs) of compounds **4b**, **4c**, **4j**, **4k**, **5b**–**d**, **5j**, **5k** were 72.4%, 55.2%, 62.1%, 75.9%, 69.0%, 62.1%, 55.2%, 65.5% and 69.0%, respectively. In particular, compound **4k** showed the most potent insecticidal activity, which was about 24% higher than toosendanin. The symptoms for the tested *M. separata* during the different periods of larval, pupation and adult were recorded by the same methods as our previous reports [19,25]. For example, in treated groups, due to overfeeding of treated leaves in the beginning, some larvae died slowly with thin and wrinkled bodies (figure 5). This phenomenon maybe results from these fraxinellone derivatives effects to nutritional or digestive interference [26]. During the pupation stage, some of the larvae did not successfully moult to normal pupae, and died (figure 6). In the last stage of emergence, many malformed moths appeared with shrunken or immature wings (figure 7). These results suggest that the fraxinellone derivatives containing the 1,3,4-oxadiazole probably affected the insect moulting hormone, which was crucial for the growth of *M. separata*. On the other hand, as displayed in figure 8, the percentages of FMRs of compounds **4b**, **4j**, **4k**, **5b**, **5c**, **5j**, **5k** and toosendanin at three different growth stages of *M. separata* were investigated. We found that at least 50% of FMRs for compounds **4b**, **4k**, **5c**, **5j**, **5k** and toosendanin were at the larval period except for compounds **4j** and **5b**.

**Table 1.** Growth inhibitory activity of compounds **1**–**3**, **4a**–**k** and **5a**–**k** against *M. separata* on leaves treated at a concentration of 1 mg ml^–1^.

	corrected mortality rate (% ± s.d.)		
compound	10 days	20 days	34 days
**1**	16.7(±3.3)	24.1(±3.3)	44.8(±3.3)
**2**	20.0(±5.8)	27.6(±5.8)	41.4(±3.3)
**3**	23.3(±3.3)	31.0(±3.3)	48.3(±5.8)
**4a**	13.3(±3.3)	24.1(±3.3)	37.9(±5.8)
**4b**	30.0(±0)	48.3(±5.8)	72.4(±6.7)
**4c**	20.0(±0)	37.9(±5.8)	55.2(±3.3)
**4d**	23.3(±3.3)	41.4(±3.3)	51.7(±3.3)
**4e**	16.7(±3.3)	31.0(±3.3)	51.7(±3.3)
**4f**	16.7(±3.3)	31.0(±3.3)	48.3(±0)
**4g**	23.3(±3.3)	31.0(±6.7)	44.8(±3.3)
**4h**	16.7(±3.3)	20.7(±3.3)	41.4(±3.3)
**4i**	13.3(±3.3)	24.1(±3.3)	37.9(±0)
**4j**	23.3(±6.7)	41.4(±3.3)	62.1(±3.3)
**4k**	33.3(±3.3)	51.7(±3.3)	75.9(±3.3)
**5a**	13.3(±3.3)	20.7(±3.3)	37.9(±5.8)
**5b**	26.7(±3.3)	41.4(±3.3)	69.0(±0)
**5c**	26.7(±3.3)	37.9(±5.8)	62.1(±3.3)
**5d**	23.3(±3.3)	34.5(±3.3)	55.2(±3.3)
**5e**	16.7(±3.3)	27.6(±0)	48.3(±5.8)
**5f**	20.0(±5.8)	31.0(±6.7)	48.3(±5.8)
**5g**	16.7(±6.7)	27.6(±0)	41.4(±3.3)
**5h**	13.3(±3.3)	17.2(±0)	34.5(±3.3)
**5i**	16.7(±3.3)	24.1(±3.3)	44.8(±3.3)
**5j**	26.7(±3.3)	41.4(±3.3)	65.5(±3.3)
**5k**	30.0(±5.8)	55.2(±3.3)	69.0(±5.8)
toosendanin	16.7(±3.3)	34.5(±3.3)	51.7(±3.3)
blank control	0(±0)	3.3(±3.3)	3.3(±3.3)

Finally, we also discovered some interesting results of structure–activity relationships of the tested compounds.

When 2'-chloroacetylfraxinellone (**2**) or 5'-chloroacetylfraxinellone (**3**) linked with 2-mercapto-5-(pyridinyl)-1,3,4-oxadiazoles exhibited more potent insecticidal activity than toosendanin. For example, the FMRs of compounds **4j**, **4 k**, **5j** and **5k** were 62.1%, 75.9%, 65.5% and 69.0%, respectively. In other fraxinellone-based thioethers, introduction of electron-donating groups on the phenyl of **4a**/**5a** resulted in less active compounds (e.g. 41.4% for **4h**, 37.9% for **4i**, 34.5% for **5h** and 44.8% for **5i**). When halogen atoms were introduced on the *para*-position of phenyl of **4a**/**5a**, the compounds were not potent (except **4e**); whereas halogen atoms at the *ortho*-position of phenyl of **4a**/**5a** gave potent compounds. For instance, the FMRs of compounds **4b**–**d** and **5b**–**d** were greater than or equal to toosendanin (51.7%), especially the FMR of **4b** was 72.4%. In our previous research, we found that introduction of heterocycle, fluorophenyl or *o*-chlorophenyl fragments on the 1,3,4-oxadiazole ring at the C-3 position of sarisan could afford more potent compounds [27]. In this paper, introduction of 3/4-pyridinyl or *o*-fluoro/chlorophenyl units on the 1,3,4-oxadiazole ring to the compound **2** or **3** also obtained the promising compounds **4j**, **5j**, **4k**, **5k**, **4b**, **5b**, **4c** and **5c**, respectively. Hence, this suggested that we could introduce the 2-mercapto-5-(3/4-pyridinyl/*o*-fluoro/chlorophenyl)-1,3,4-oxadiazoles activity units into other insecticidal lead compounds in the future.

## 4. Conclusion

In summary, we have prepared two series of novel fraxinellone-based thioethers containing 1,3,4-oxadiazole and evaluated for their insecticidal activity against a cereal crop-threatening agricultural insect pest, *M. separata*. The structures of key compounds **4e** and **5d** were assigned by X-ray crystallography. Among all target compounds, compounds **4b**, **4k**, **5b**, **5j** and **5k** exhibited more potent insecticidal activity with FMRs of more than 65%. The results suggested that the introduction of 3/4-pyridinyl or *o*-fluoro/chlorophenyl units on the 1,3,4-oxadiazole ring to the compound **2** or **3** could afford more promising compounds. This will lay the foundations for further structural modification and application of fraxinellone as novel pesticidal agents in agriculture.

## Supplementary Material

Supporting Information

## Data accessibility

Electronic supplementary material is available at the Dryad Digital Repository: https://doi.org/10.5061/dryad.424tb [28]. Crystallographic data (excluding structure factors) of compounds **4e** and **5d** were deposited at the Cambridge Crystallographic Data Centre (CCDC) with deposition numbers of CCDC 1552786 and 1552787, respectively. Copies of the data can be obtained, free of charge, on application to CCDC, 12 Union Road, Cambridge CB2 1EZ, U.K. [fax, +44 (0)1223 336033; or e-mail, deposit@ccdc.cam.ac.uk].
