# Supplementary material for: Semisynthesis and insecticidal activity of some novel fraxinellone-based thioethers containing 1,3,4-oxadiazole moiety
Source: R Soc Open Sci. 2017 Dec 13;4(12):171053. doi: 10.1098/rsos.171053 (PMC5750010; doi:10.1098/rsos.171053)
Supplement: Supporting Information [file rsos171053supp1.doc]

Supporting Information

**Semisynthesis and insecticidal activity of some novel fraxinellone-based thioethers containing 1,3,4-oxadiazole moiety**

Yong Guo,^a^,* Xiaoguang Wang,^a^ Jiangping Fan,^a^ Qian Zhang,^a^ Yi Wang,^b^ Yi Zhao,^a^ Mengxing Huang,^a^ Ming Ding,^a^ Yanbing Zhang^a^,*

*^a^* *Key Laboratory of Advanced Drug Preparation Technologies, Ministry of Education, School of Pharmaceutical Sciences, Zhengzhou University, No. 100, KeXue Avenue, Zhengzhou, 450001, Henan Province, PR China*

*^b^College of Agriculture, Shanxi Agriculture University, Taigu 030801, Shanxi Province, PR China*

*Corresponding authors: Tel. /fax: +86 371 67781912, e–mail: guoyong_122@zzu.edu.cn; zhangyb@zzu.edu.cn.

**Spectra data:**

*Data for* ***4e***: White solid, yield: 73%, m.p. 150–152 ^o^C; [α]^20^_D_ = 15 (*c* 3.4 mg/mL, acetone); IR cm^–1^: 2964, 2938, 1740, 1674, 1609, 1500, 1476, 1413, 1207, 1156, 1049; ^1^H NMR (400 MHz, CDCl_3_) *δ*: 7.98 (dd, *J* = 8.8, 1.2 Hz, 2H, –Ph), 7.60 (d, *J* = 1.2 Hz, 1H, H–5′), 7.17 (t, *J* = 8.4 Hz, 2H, –Ph), 6.83 (d, *J* = 1.2 Hz, 1H, H–4′), 5.66 (s, 1H, H–8), 4.68–4.86 (m, 2H, –COC*H*_2_S–), 2.13–2.26 (m, 5H, H–4, 10), 1.71–1.78 (m, 2H, H–5, 6), 1.55–1.59 (m, 2H, H–5, 6), 0.86 (s, 3H, H–11).

*Data for* ***4f***: Pale yellow solid, yield: 77%, m.p. 164–167 ^o^C; [α]^20^_D_ = 8 (*c* 3.0 mg/mL, acetone); IR cm^–1^: 2936, 1743, 1676, 1606, 1472, 1466, 1413, 1260, 1207, 1130, 1049; ^1^H NMR (400 MHz, CDCl_3_) *δ*: 7.92 (d, *J* = 8.4 Hz, 2H, –Ph), 7.60 (d, *J* = 1.6 Hz, 1H, H–5′), 7.46 (d, *J* = 8.8 Hz, 2H, –Ph), 6.83 (d, *J* = 1.6 Hz, 1H, H–4′), 5.66 (s, 1H, H–8), 4.68–4.87 (m, 2H, –COC*H*_2_S–), 2.15–2.26 (m, 2H, H–4), 2.13 (s, 3H, H–10), 1.71–1.77 (m, 2H, H–5, 6), 1.50–1.59 (m, 2H, H–5, 6), 0.86 (s, 3H, H–11).

*Data for* ***4g***: White solid, yield: 59%, m.p. 130–132 ^o^C; [α]^20^_D_ = 1 (*c* 2.0 mg/mL, acetone); IR cm^–1^: 2935, 1746, 1674, 1601, 1470, 1414, 1353, 1275, 1206, 1074; ^1^H NMR (400 MHz, CDCl_3_) *δ*: 7.84 (d, *J* = 8.4 Hz, 2H, –Ph), 7.63 (d, *J* = 8.4 Hz, 2H, –Ph), 7.60 (d, *J* = 1.6 Hz, 1H, H–5′), 6.83 (d, *J* = 1.6 Hz, 1H, H–4′), 5.66 (s, 1H, H–8), 4.68–4.86 (m, 2H, –COC*H*_2_S–), 2.08–2.26 (m, 5H, H–4, 10), 1.71–1.77 (m, 2H, H–5, 6), 1.54–1.58 (m, 2H, H–5, 6), 0.86 (s, 3H, H–11).

*Data for* ***4h***: White solid, yield: 78%, m.p. 117–119 ^o^C; [α]^20^_D_ = 7 (*c* 3.3 mg/mL, acetone); IR cm^–1^: 2922, 1750, 1672, 1587, 1473, 1416, 1357, 1204, 1131, 1046; ^1^H NMR (400 MHz, CDCl_3_) *δ*: 7.84 (d, *J* = 7.6 Hz, 1H, –Ph), 7.59 (d, *J* = 1.6 Hz, 1H, H–5′), 7.39–7.43 (m, 1H, –Ph), 7.31–7.34 (m, 2H, –Ph), 6.82 (d, *J* = 1.6 Hz, 1H, H–4′), 5.67 (s, 1H, H–8), 4.66–4.86 (m, 2H, –COC*H*_2_S–), 2.67 (s, 3H, –C*H*_3_), 2.13–2.26 (m, 5H, H–4, 10), 1.71–1.77 (m, 2H, H–5, 6), 1.56–1.65 (m, 2H, H–5, 6), 0.86 (s, 3H, H–11).

*Data for* ***4i***: White solid, yield: 68%, m.p. 171–172 ^o^C; [α]^20^_D_ = 2 (*c* 3.8 mg/mL, acetone); IR cm^–1^: 2923, 1751, 1672, 1612, 1590, 1503, 1475, 1261, 1205, 1172, 1048, 1023; ^1^H NMR (400 MHz, CDCl_3_) *δ*: 7.91 (d, *J* = 8.8 Hz, 2H, –Ph), 7.59 (d, *J* = 1.6 Hz, 1H, H–5′), 6.97 (d, *J* = 9.2 Hz, 2H, –Ph), 6.82 (d, *J* = 1.2 Hz, 1H, H–4′), 5.67 (s, 1H, H–8), 4.64–4.84 (m, 2H, –COC*H*_2_S–), 3.87 (s, 3H, H–11), 2.13–2.26 (m, 5H, H–4, 10), 1.71–1.77 (m, 2H, H–5, 6), 1.55–1.60 (m, 2H, H–5, 6), 0.86 (s, 3H, H–11).

*Data for* ***4j***: White solid, yield: 56%, m.p. 165–167 ^o^C; [α]^20^_D_ = 18 (*c* 3.2 mg/mL, acetone); IR cm^–1^: 2947, 2914, 1754, 1672, 1586, 1471, 1415, 1356, 1204, 1062, 1046; ^1^H NMR (400 MHz, CDCl_3_) *δ*: 8.80 (d, *J* = 5.6 Hz, 2H, pyridine ring), 7.84 (d, *J* = 6.0 Hz, 2H, pyridine ring), 7.62 (d, *J* = 1.6 Hz, 1H, H–5′), 6.84 (d, *J* = 1.6 Hz, 1H, H–4′), 5.66 (s, 1H, H–8), 4.72–4.90 (m, 2H, –COC*H*_2_S–), 2.09–2.27 (m, 5H, H–4, 10), 1.70–1.77 (m, 2H, H–5, 6), 1.55–1.61 (m, 2H, H–5, 6), 0.87 (s, 3H, H–11).

*Data for* ***4k***: White solid, yield: 96%, m.p. 79–80 ^o^C; [α]^20^_D_ = 9 (*c* 3.8 mg/mL, acetone); IR cm^–1^: 2936, 1753, 1672, 1584, 1470, 1413, 1357, 1279, 1204, 1046, ; ^1^H NMR (400 MHz, CDCl_3_) *δ*: 9.22 (d, *J* = 1.6 Hz, 1H, pyridine ring), 8.76 (dd, *J* = 4.8, 1.2 Hz, 1H, pyridine ring), 8.28 (dt, *J* = 8.0, 1.6 Hz, 1H, pyridine ring), 7.61 (d, *J* = 1.6 Hz, 1H, H–5′), 7.45 (dd, *J* = 8.0, 4.8 Hz, 1H, pyridine ring), 6.83 (d, *J* = 1.6 Hz, 1H, H–4′), 5.66 (s, 1H, H–8), 4.70–4.89 (m, 2H, –COC*H*_2_S–), 2.15–2.26 (m, 2H, H–4 ), 2.13 (s, 3H, H–10), 1.71–1.74 (m, 2H, H–5, 6), 1.55–1.63 (m, 2H, H–5, 6), 0.87 (s, 3H, H–11).

*Data for* ***5e***: White solid, yield: 76%, m.p. 198–200 ^o^C; [α]^20^_D_ = –8 (*c* 2.6 mg/mL, acetone); IR cm^–1^: 2962, 2922, 1745, 1674, 1608, 1502, 1479, 1205, 1167, 1002; ^1^H NMR (400 MHz, CDCl_3_) *δ*: 7.98–8.02 (m, 2H, –Ph), 7.71 (s, 1H, H–2′), 7.29 (s, 1H, H–4′), 7.17 (t, *J* = 8.8 Hz, 2H, –Ph), 4.90 (s, 1H, H–8), 4.73 (s, 2H, –COC*H*_2_S–), 2.17–2.34 (m, 2H, H–4), 2.14 (s, 3H, H–10), 1.73–1.87 (m, 3H, H–5, 6), 1.46–1.54 (m, 1H, H–6), 0.85 (s, 3H, H–11).

*Data for* ***5f***: White solid, yield: 82%, m.p. 160–162 ^o^C; [α]^20^_D_ = –18 (*c* 3.3 mg/mL, acetone); IR cm^–1^: 2934, 2869, 1746, 1686, 1598, 1511, 1464, 1309, 1211, 1162, 1073; ^1^H NMR (400 MHz, CDCl_3_) *δ*: 7.92 (d, *J* = 8.8 Hz, 2H, –Ph), 7.71 (s, 1H, H–2′), 7.46 (d, *J* = 8.8 Hz, 2H, –Ph), 7.29 (s, 1H, H–4′), 4.89 (s, 1H, H–8), 4.73 (s, 2H, –COC*H*_2_S–), 2.17–2.34 (m, 2H, H–4), 2.14 (s, 3H, H–10), 1.73–1.87 (m, 3H, H–5, 6), 1.46–1.54 (m, 1H, H–6), 0.85 (s, 3H, H–11).

*Data for* ***5g***: White solid, yield: 91%, m.p. 178–180 ^o^C; [α]^20^_D_ = –5 (*c* 2.9 mg/mL, acetone); IR cm^–1^: 2934, 2869, 1746, 1686, 1598, 1512, 1481, 1463, 1212, 1162, 1073; ^1^H NMR (400 MHz, CDCl_3_) *δ*: 7.85 (d, *J* = 8.4 Hz, 2H, –Ph), 7.71 (s, 1H, H–2′), 7.63 (d, *J* = 8.8 Hz, 2H, –Ph), 7.29 (s, 1H, H–4′), 4.89 (s, 1H, H–8), 4.73 (s, 2H, –COC*H*_2_S–), 2.18–2.34 (m, 2H, H–4), 2.14 (s, 3H, H–10), 1.73–1.87 (m, 3H, H–5, 6), 1.46–1.53 (m, 1H, H–6), 0.85 (s, 3H, H–11).

*Data for* ***5h***: White solid, yield: 52%, m.p. 144–146 ^o^C; [α]^20^_D_ = –10 (*c* 3.8 mg/mL, acetone); IR cm^–1^: 2928, 1746, 1676, 1600, 1508, 1470, 1392, 1318, 1208, 1169, 1048; ^1^H NMR (400 MHz, CDCl_3_) *δ*: 7.85 (d, *J* = 8.0 Hz, 1H, –Ph), 7.70 (s, 1H, H–2′), 7.39–7.43 (m, 1H, –Ph), 7.29–7.34 (m, 3H, –Ph and H–4′), 4.89 (s, 1H, H–8), 4.72 (s, 2H, –COC*H*_2_S–), 2.67 (s, 3H, –C*H*_3_), 2.18–2.34 (m, 2H, H–4), 2.14 (s, 3H, H–10), 1.72–1.90 (m, 3H, H–5, 6), 1.46–1.53 (m, 1H, H–6), 0.85 (s, 3H, H–11).

*Data for* ***5i***: White solid, yield: 93%, m.p. 180–182 ^o^C; [α]^20^_D_ = –8 (*c* 3.1 mg/mL, acetone); IR cm^–1^: 2941, 1747, 1686, 1613, 1502, 1473, 1306, 1253, 1164, 1029; ^1^H NMR (400 MHz, CDCl_3_) *δ*: 7.91 (d, *J* = 8.8 Hz, 2H, –Ph), 7.70 (s, 1H, H–2′), 7.29 (s, 1H, H–4′), 6.98 (d, *J* = 8.8 Hz, 2H, –Ph), 4.89 (s, 1H, H–8), 4.70 (s, 2H, –COC*H*_2_S–), 3.87 (s, 3H, –OC*H*_3_), 2.17–2.34 (m, 2H, H–4), 2.14 (s, 3H, H–10), 1.72–1.87 (m, 3H, H–5, 6), 1.47–1.52 (m, 1H, H–6), 0.85 (s, 3H, H–11).

*Data for* ***5j***: Pale yellow solid, yield: 59%, m.p. 173–175 ^o^C; [α]^20^_D_ = –15 (*c* 3.7 mg/mL, acetone); IR cm^–1^: 2963, 2939, 2920, 1753, 1672, 1468, 1401, 1201, 1168, 1133, 1046; ^1^H NMR (400 MHz, CDCl_3_) *δ*: 8.80 (d, *J* = 4.4 Hz, 2H, pyridine ring), 7.86 (d, *J* = 6.0 Hz, 2H, pyridine ring), 7.72 (s, 1H, H–2′), 7.30 (s, 1H, H–4′), 4.90 (s, 1H, H–8), 4.77 (s, 2H, –COC*H*_2_S–), 2.17–2.34 (m, 2H, H–4), 2.15 (s, 3H, H–10), 1.72–1.87 (m, 3H, H–5, 6), 1.46–1.54 (m, 1H, H–6), 0.86 (s, 3H, H–11).

*Data for* ***5k***: Pale yellow solid, yield: 54%, m.p. 124–126 ^o^C; [α]^20^_D_ = –15 (*c* 4.1 mg/mL, acetone); IR cm^–1^: 2964, 2940, 2927, 1750, 1670, 1598, 1471, 1395, 1307, 1205, 1168, 1047; ^1^H NMR (400 MHz, CDCl_3_) *δ*: 9.23 (s, 1H, pyridine ring), 8.77 (d, *J* = 2.8 Hz, 1H, pyridine ring), 8.30 (d, *J* = 8.0 Hz, 1H, pyridine ring), 7.71 (s, 1H, H–2′), 7.46 (dd, *J* = 8.0, 4.8 Hz, 1H, pyridine ring), 7.30 (s, 1H, H–4′), 4.90 (s, 1H, H–8), 4.75 (s, 2H, –COC*H*_2_S–), 2.19–2.34 (m, 2H, H–4), 2.14 (s, 3H, H–10), 1.73–1.91 (m, 2H, H–5, 6), 1.46–1.54 (m, 1H, H–6), 0.86 (s, 3H, H–11).
